# 3D landscape of Hepatitis B virus interactions with human chromatins

**DOI:** 10.1038/s41421-020-00218-1

**Published:** 2020-12-29

**Authors:** Bo Yang, Boyuan Li, Liyang Jia, Yongpeng Jiang, Xin Wang, Shaodong Jiang, Shunda Du, Xiong Ji, Pengyuan Yang

**Affiliations:** 1 CAS Key Laboratory of Infection and Immunity, CAS Center for Excellence in Biomacromolecules, Institute of Biophysics, University of Chinese Academy of Sciences, Chinese Academy of Sciences, Beijing, 100101 China; 2 Key Laboratory of Cell Proliferation and Differentiation of the Ministry of Education, School of Life Sciences, Peking-Tsinghua Center for Life Sciences, Peking University, Beijing, 100871 China; 3 Department of Liver Surgery, Peking Union Medical College (PUMC) Hospital, Chinese Academy of Medical Science and PUMC, Beijing, 100730 China

**Keywords:** Chromatin structure, Nuclear organization

## Abstract

Hepatitis B viral (HBV) DNAs, including covalently closed circular DNA (cccDNA) and integrated HBV DNA forms, are considered to be primary contributors to the development and progression of HBV-associated liver diseases. However, it remains largely unclear how HBV DNAs communicate with human chromatin. Here we employed a highly sensitive technology, 3C-high-throughput genome-wide translocation sequencing (3C-HTGTS), to globally identify HBV DNA–host DNA contacts in cellular models of HBV infection. HBV DNA does not randomly position in host genome but instead preferentially establishes contacts with the host DNA at active chromatin regions. HBV DNA–host DNA contacts are significantly enriched at H3K4me1-marked regions modified by KMT2C/D; this histone modification is also observed in the HBV cccDNA mini-chromosome and strongly influences HBV transcription. On the other hand, chromatin loop formed by integrated HBV DNA with host genomic DNA was found in transcriptionally active regions. Furthermore, HBV infection influences host gene expression accompanied with HBV DNA–host DNA contacts. Our study provides a 3D landscape of spatial organization of cccDNA and integrated HBV DNA within the human genome, which lays the foundation for a better understanding of the mechanisms how HBV involves in liver disease development and progression.

## Introduction

About 240 million people worldwide are chronically infected with Hepatitis B virus (HBV), which remains a serious global health problem^1,2^. Uncontrolled chronic HBV infection can progress to life-threatening end-stage chronic liver diseases, such as cirrhosis and hepatocellular carcinoma. HBV is a small enveloped DNA virus of 3.2 kb, which belongs to the Hepadnaviride family, and replicates through the reverse transcription of pregenomic RNA (pgRNA) into relaxed circular partially double-stranded DNA (rcDNA)^3^. Upon internalization and transfer to the nucleus, the viral rcDNA is converted to covalently closed circular DNA (cccDNA), which is the bona fide viral transcription template and considered as the main contributor to chronic infection. Moreover, the HBV DNA can integrate into the genomes of infected liver cells, which progressively contributes to hepatocarcinogenesis. However, although the HBV DNA long-term persists within human nucleus and contributes to malignant liver diseases, it remains unclear yet how the HBV DNAs communicate with human chromatin.

In the liver cell nucleus, the cccDNA assembles together with host cellular histone proteins into episomal mini-chromatin^4^, which is dynamically regulated by posttranslational machinery modifications (PTMs) of histone proteins to support the viral gene expression^5,6^. Although previous studies mapped the PTMs in cccDNA-containing chromatin and identified H3K4me3 and other active modifications enriched in HBV promoters^7^, it remains unclear which and how PTMs are dominantly enriched at the contact regions with host genome. In contrast, the integrated HBV DNA only expresses viral S antigen but is supposed to induce a diverse collection of genomic perturbations near viral integration sites, including direct gene disruption and viral promoter-driven host gene transcription. Although a recent study starts to explore viral DNA capture Hi-C to investigate the spatial nuclear organization of DNA^8^, the HBV DNA organization in human nuclear are still obscure due to the sensitivity of the detection method, especially the integrated HBV DNA.

In this study, we conducted high-throughput genome-wide translocation sequencing-coupled chromosome conformation capture (HTGTS-3C)^9^, a highly sensitive method to identify the regions interacting with HBV DNA genomewide. For this method, the 3C library with a 4 bp cutting restriction endonuclease and linear amplification-mediated HTGTS were exploited to generate highly sensitive and specific interaction profiles for widely separated bait and prey sequences. Two kinds of HBV cellular models, including an HBV-infected HepG2-NTCP cells and HBV-integrated cell line HepAD38, were employed for the 3C-HTGTS analyses. Consistent with previous report, HBV DNA interacts with the whole host genome, strongly correlated with active histone modifications (H3K4me3, H3K9ac, and H3K27ac) and DNA elements associated with active transcription (transcription start site (TSS), promoter, and CpG islands). Interestingly, our data indicated HBV–host DNA contacts are significantly enriched in enhancers that are associated with H3K4me1, which positively regulates HBV transcription and is dependent on the activity of KMT2C/D. In addition, the integrated HBV DNAs organize chromatin loop structures linking active host DNA, adjacent to the integration sites. Our study provided a regulatory landscape of the spatial organizations of HBV DNA and integrated DNA within human genome, which serves as the molecular basis for shared epigenetic machineries between HBV virus and host genome.

## Results

### Genome-wide organization of HBV DNA contacts at the host genome

**Fig. 1:**
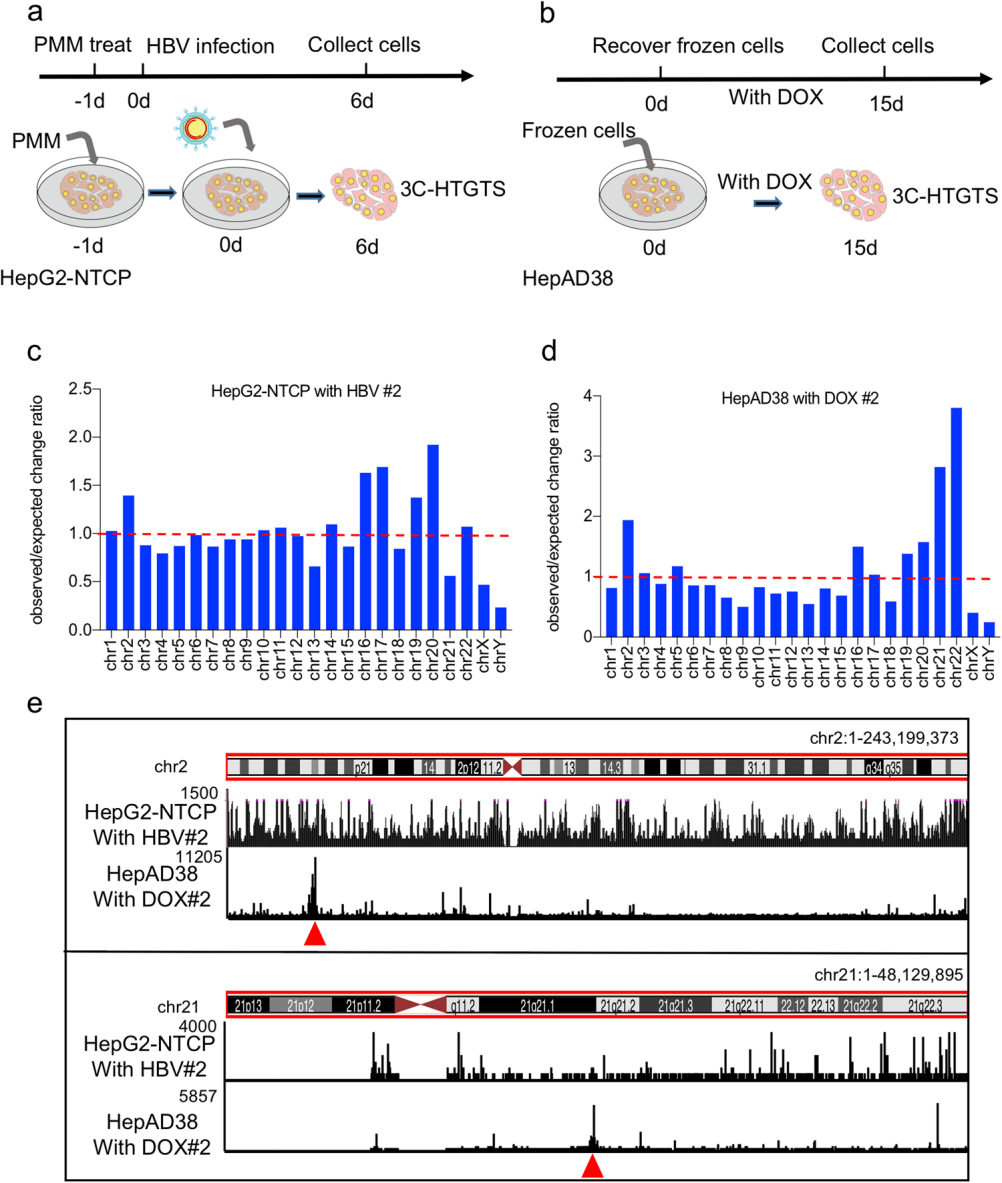
Distribution of HBV DNA–host DNA contacts throughout the Human genome. **a**, **b** Model of the cell treatment in HBV infection HepG2-NTCP cell system (**a**) and inducible expression of HBV HepAD38 cell system (**b**). The HepG2-NTCP cells after HBV infection 6 days and the HepAD38 cells treated with DOX 15 days were used to carry out the 3C-HTGTS experiments. **c**, **d** The HBV DNA–host DNA contacts distribution density in different chromosome. HBV infected HepG2-NTCP cells using primer 2 (**c**) and HepAD38 with DOX cells using primer 2 (**d**). **e** UCSC Genome Browser track showing HBV DNA–host DNA contacts throughout the human genome in HepG2-NTCP cell model and in HepAD38 with DOX cell model, respectively (we used vertical viewing range setting for sequencing data display). The red arrows indicate the HBV DNA integration site in HepAD38 cell.

To detect HBV DNA contacts across the host genome, we employed 3C-HTGTS to genome-wide sequencing all regions that contact with the target DNA. As shown in the Supplementary Fig. S1a, we first fixed the cells with formaldehyde to cross-link proteins to proteins and to DNA. Cross-linked chromatin was subsequently digested with restriction enzyme and chromatin fragments were re-ligated in situ. The DNA was reversed cross-linked, purified, and then amplified with LAM-PCR. After adapter ligation with a bridged linker, we used nested PCR to amplify the ligated DNA products for the next-generation sequencing. The AluI restriction enzyme was selected based on the efficiency of chromatin digestion and the primers that we designed for testing the HBV DNA–host DNA contacts (Supplementary Fig. S1b-d). To investigate the spatial organization of HBV DNA in host nuclear, two HBV cell models were employed including HBV-infected HepG2-NTCP cells^10^ and the HepAD38 cell line harboring a tetracycline-controlled Tet-Off system, silencing integrated HBV genome expression by tetracycline derivatives such as doxycycline (DOX)^11^. As shown in Supplementary Fig. S2a-d, the viral RNAs and cccDNA of HBV were highly amplified at day 6 after infection of HepG2-NTCP cells and HepAD38 cells with DOX, indicating the validity of the two HBV cellular models. We collected the HBV-infected HepG2-NTCP cells as a de novo infection stage and HepAD38 cells with DOX as an HBV genome integrated model to launch the 3C-HTGTS determinations (Fig. 1a, b). The HepG2-NTCP cells were collected at 6 days after HBV infection and the HepAD38 cells that were treated with DOX constitutively. To avoid the bias caused by primer preference, we designed two different primers (1# and 2#) for 3C-HTGTS to get high-confident interaction sites. We total got specific HBV DNA–host DNA contacts 58,819 (primer I) and 79,315 (primer II) in HBV-infected HepG2-NTCP cells, as well as 89,070 (primer I) and 78,536 (primer II) in HepAD38 cells, respectively (Supplementary Fig. S2e). With the negative control primer, we totally got 857,567 reads; after selection, there were only 34 reads. We found that HBV DNA contacts with the host genome and the HBV DNA–host DNA contacts covered different chromosomes with different density (Fig. 1c-e and Supplementary Fig. S2f). Especially, HBV DNA–host DNA contacts in chromosomes 13, 15, 18, 21, X, Y with lower density and chromosomes 16, 17, 19, 20 with higher density in HepG2-NTCP with HBV #2 (Fig. 1c), indicating that HBV DNA contacts with host DNA non-randomly.

### HBV DNA contacts host genome preferentially at active chromatin regions

**Fig. 2:**
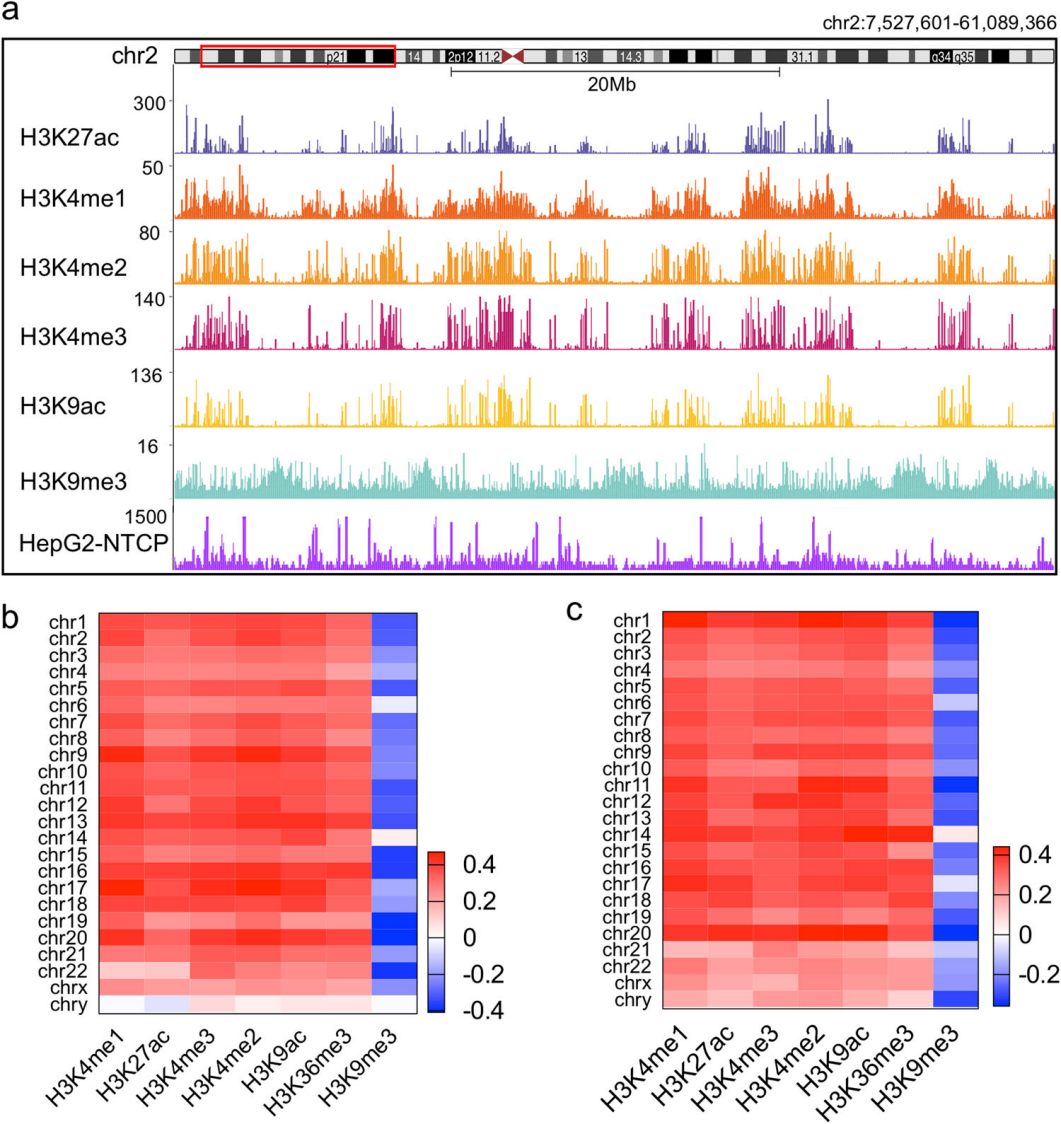
HBV DNA–host DNA contacts are preferential at active chromatin regions. **a** UCSC Genome Browser track showing HBV DNA–host DNA contacts are preferential at the H3K27ac, H3K4me3, and H3K9ac marked region, and are not enriched at the H3K9me3 marked regions (using HepG2-NTCP #2 3C-HTGTS sample and we used vertical viewing range setting for sequencing data display). **b**, **c** Heatmap of HBV DNA–host DNA contacts enriched in different histone marks in different chromosomes. Signals for the heatmap are represented by Spearman correlation coefficient. Public available histone mark ChIP-Seq datasets were from HepG2 cells. HBV infected HepG2-NTCP cells using primer 2 (**b**) and primer 1 (**c**).

**Fig. 3:**
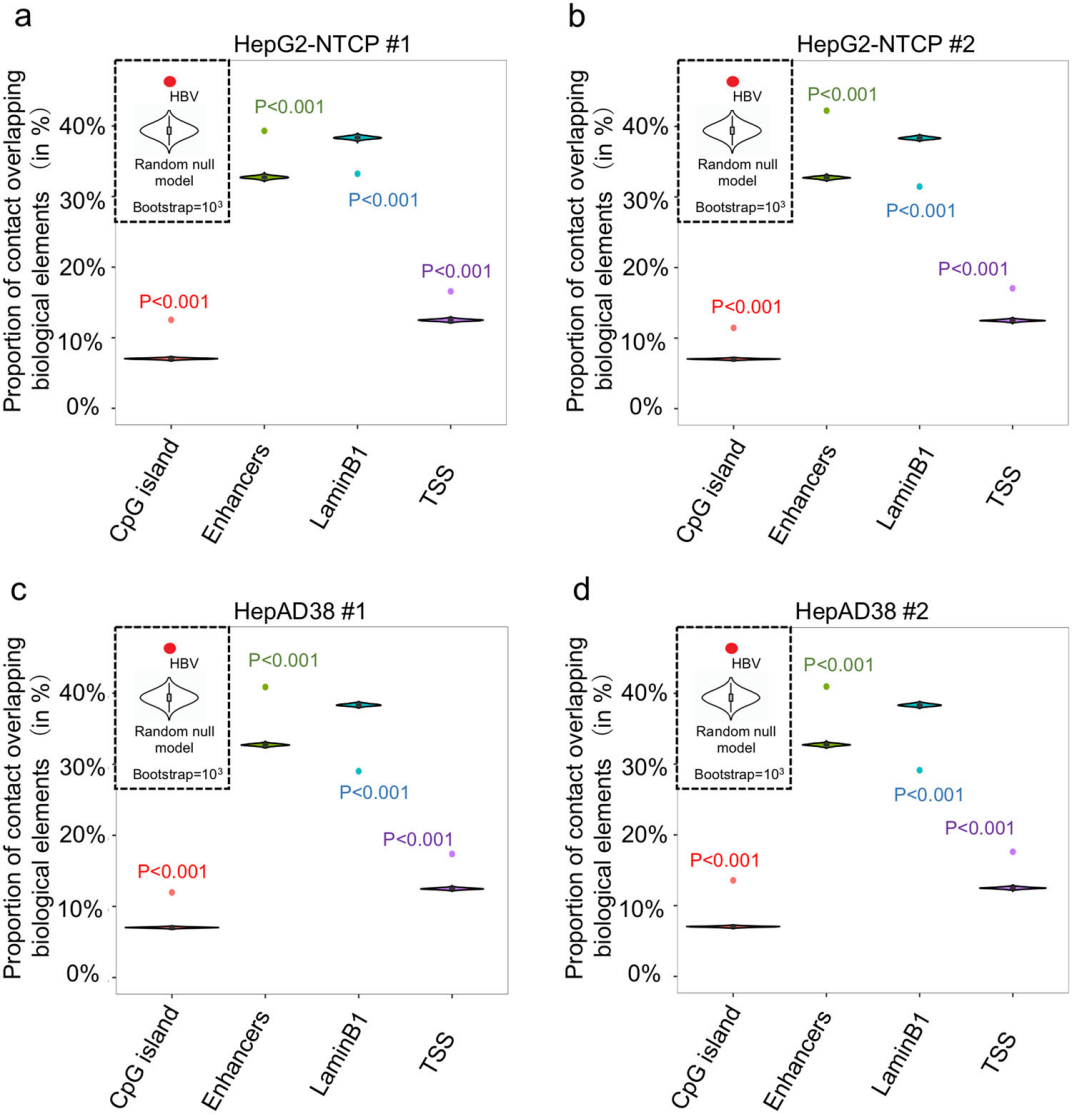
HBV DNA–host DNA contacts are preferential at CpG island, enhancers, and TSS regions. **a**–**d** The proportions of HBV DNAs contact with different elements; violin and box plot represent the distribution of the proportions of random select reads (±3.5 kb around the Alu1 enzyme sites) overlapping with each element; point represents the proportions of HBV contact reads (±3.5 kb around the start site of the read) overlapping with each element. Top left violin inserts representing the random sample model followed the Ref. ^29^.

HBV cccDNA assembled with cellular histone proteins into chromatin and its transcription depends on the cellular transcriptional machinery. Histone in HBV cccDNA mini-chromosome was marked by H3K4me2, H3K4me3, H3K9ac, H3K27ac, and H3K36me3^5–7,12^, which were associated with active transcription, but less modified by H3K9me3, which indicated silent regions. Therefore, we hypothesize that HBV preferentially interacts with the active regions of host genome, which may share similar epigenetic histone marks with cccDNA. Previous studies have indicated similarly modified regions were usually constrained with ~1 Mb topologically associating domains; thus, a 250 kb binning to characterize contacts between the HBV DNA and host DNA was selected. At this resolution, HBV cccDNA–host DNA contacts enriched in the chromatin regions that were marked by H3K4me2, H3K4me3, H3K9ac, H3K27ac, and H3K36me3 (Fig. 2a-c and Supplementary Fig. S3a-c). At 50 kb resolution, HBV DNA–host DNA contact was similar to that at 250 kb resolution (Supplementary Fig. S3d). H3K4me3 was found at the TSSs of actively transcribed genes, whereas histone H3K27ac modification indicates active promoters and enhancers. In addition, CpG island mainly associated with the promoter region. Thus, we tested the correlation between HBV DNA–host DNA contact with the DNA elements of TSSs, enhancers and CpG islands. We found that HBV DNA–host DNA contact enriched at TSSs, enhancers, and CpG islands (Fig. 3a-d). However, HBV DNA–host DNA contacts did not enrich at LaminB1 sites, indicating that HBV DNA did not interact with the repressive nuclear envelope (Fig. 3a-d). Taken together, our results indicate that HBV DNA contacts with host genome at active chromatin regions, providing a three-dimensional framework for sharing epigenetic regulators.

### KMT2C/D-modified H3K4me1 is required for HBV transcription

**Fig. 4:**
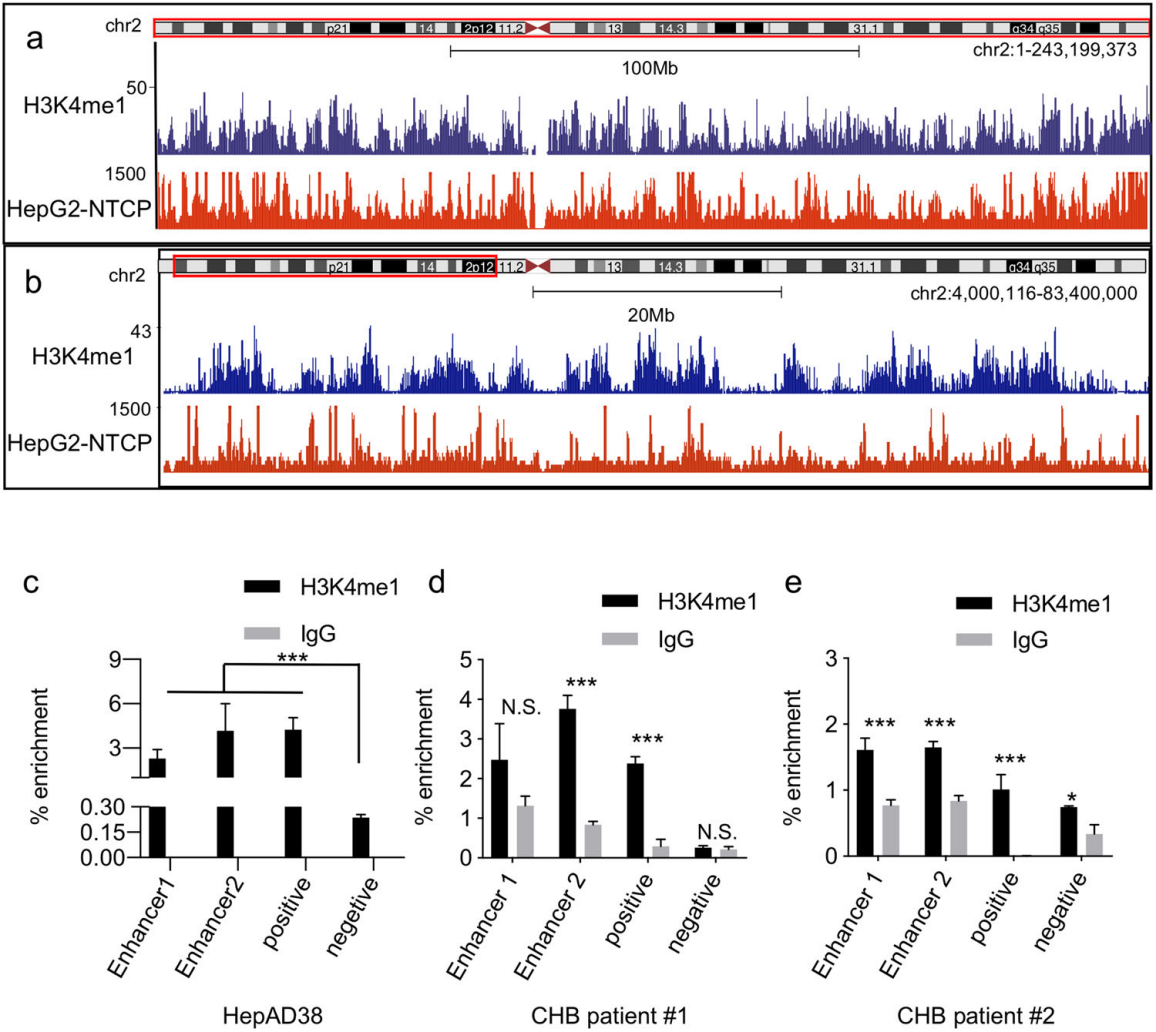
Histone posttranslational modifications H3K4me1 in HBV cccDNA. **a**, **b** UCSC Genome Browser track (using HepG2-NTCP #2 3C-HTGTS sample and we used vertical viewing range setting for sequencing data display) showing HBV DNA–host DNA contact are preferential at the H3K4me1 region in chromosome 2 (**a**) and in a small region of chromosome 2 (**b**). **c** H3K4me1 recruitment was analyzed by ChIP-qPCR in HepAD38 cells. The primer regions are the HBV genome Enhancer-I, enhancer-II, and Human genome positive regions and negative regions. **d**, **e** The ChIP assay performed in human liver samples. Error bars indicate SEM and an asterisk a significant statistical difference (*P < 0.05, ***P < 0.001).

As expected, HBV DNA–host DNA contacts are enriched in the region marked by H3K9ac, H3K4me3, and H3K27ac. Interestingly, the H3K4me1 was also found to be enriched within the HBV DNA–host DNA contact regions (Figs. 2a-c and 4a, b), which has not been reported in HBV cccDNA previously. We then employed chromatin immunoprecipitation-quantitative PCR (ChIP-qPCR) method to investigate whether this H3K4me1 modification exists in HBV DNA or not. As shown in Fig. 4c and Supplementary Fig. S4a, b, H3K4me1 modification was detected at the HBV cccDNA, which support our hypothesis that HBV mini-chromosome shares the histone modifications with contacting regions of host genome. The histone H3K4me1 modification was also found in HBV-infected patients and was more nuanced than observed in in vitro models (Fig. 4d, e and Supplementary Fig. S4c).

**Fig. 5:**
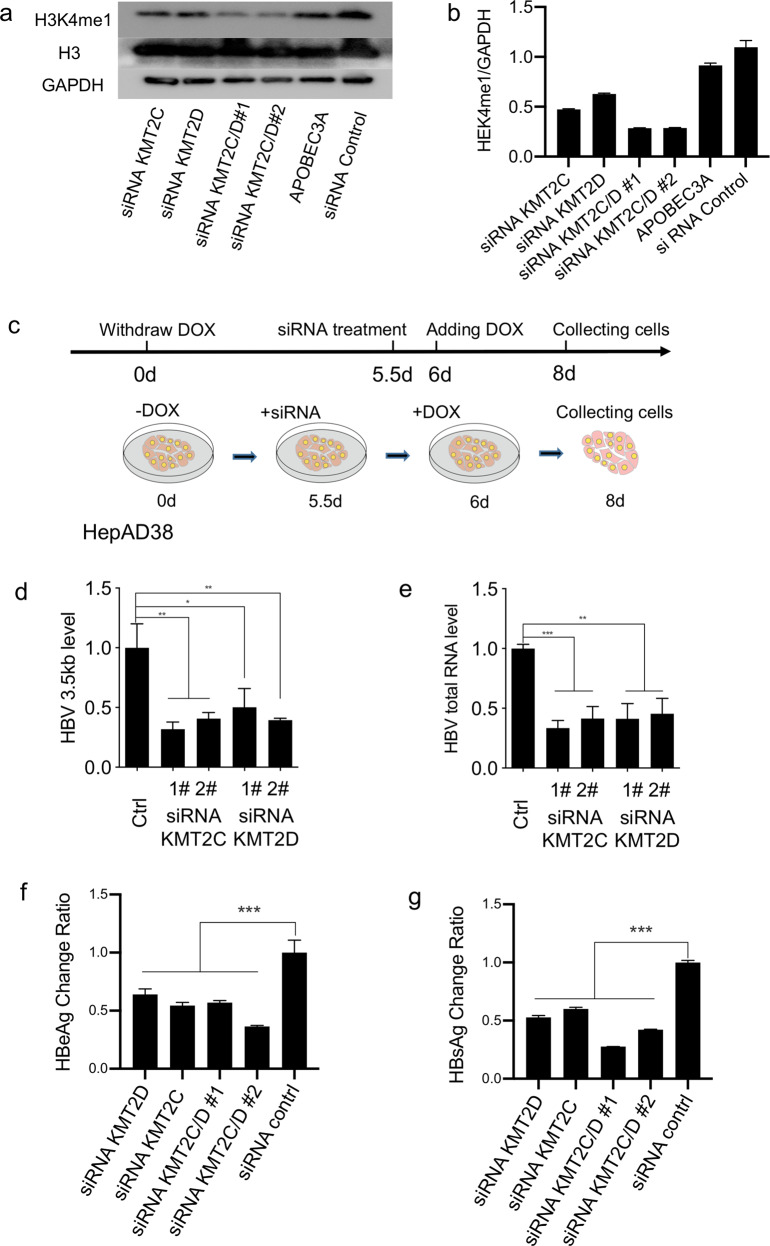
KMT2C/D are required for HBV transcription. **a**, **b** The effect of KMT2C/D knockdown on H3K4me1 protein level. **a** The result of western blotting. **b** The quantitative statistics for the western blot result by Image J. **c** The model of treatment in HepAD38 cells, the DOX were re-added after siRNA transfection for 12 h, and the cells were collected for HBV RNAs detection 2.5 days post siRNA transfection. **d**, **e** HBV transcription was analyzed by RT-qPCR in HepAD38 transfected with siRNA. **f**, **g** HBeAg and HBsAg were analyzed by ELISA in HepG2, which were transfected by HBV plasmid and siRNA. Error bars indicate SEM and an asterisk a significant statistical difference (*P < 0.05, **P < 0.01, ***P < 0.001).

KMT2C and KMT2D were known as key methyltransferases for H3K4me1^13,14^; we next investigated the potential role of H3K4me1 in HBV DNA transcription and replication with small interfering RNA knockdown of KMT2C/D in HepAD38 cells. Both KMT2C and KMT2D knockdown markedly suppressed the level of HBV total RNAs and 3.5 kb RNA (Fig. 5a-e) in HepAD38 cells. Importantly, the protein expression of HBsAg and HBeAg was also significantly inhibited by KMT2C or KMT2D knockdown (Fig. 5f, g). However, the expression of HBV mRNA and HBeAg were not suppressed by KMT2C/D knockdown before HBV infection 2.5 days in HepG2-NTCP cells (Supplementary Fig. S5a-h). Taken together, our data demonstrates that H3K4me1 is enriched at HBV genome and required for HBV transcription.

### The genome organizations of integrated HBV DNA within human chromosomes

**Fig. 6:**
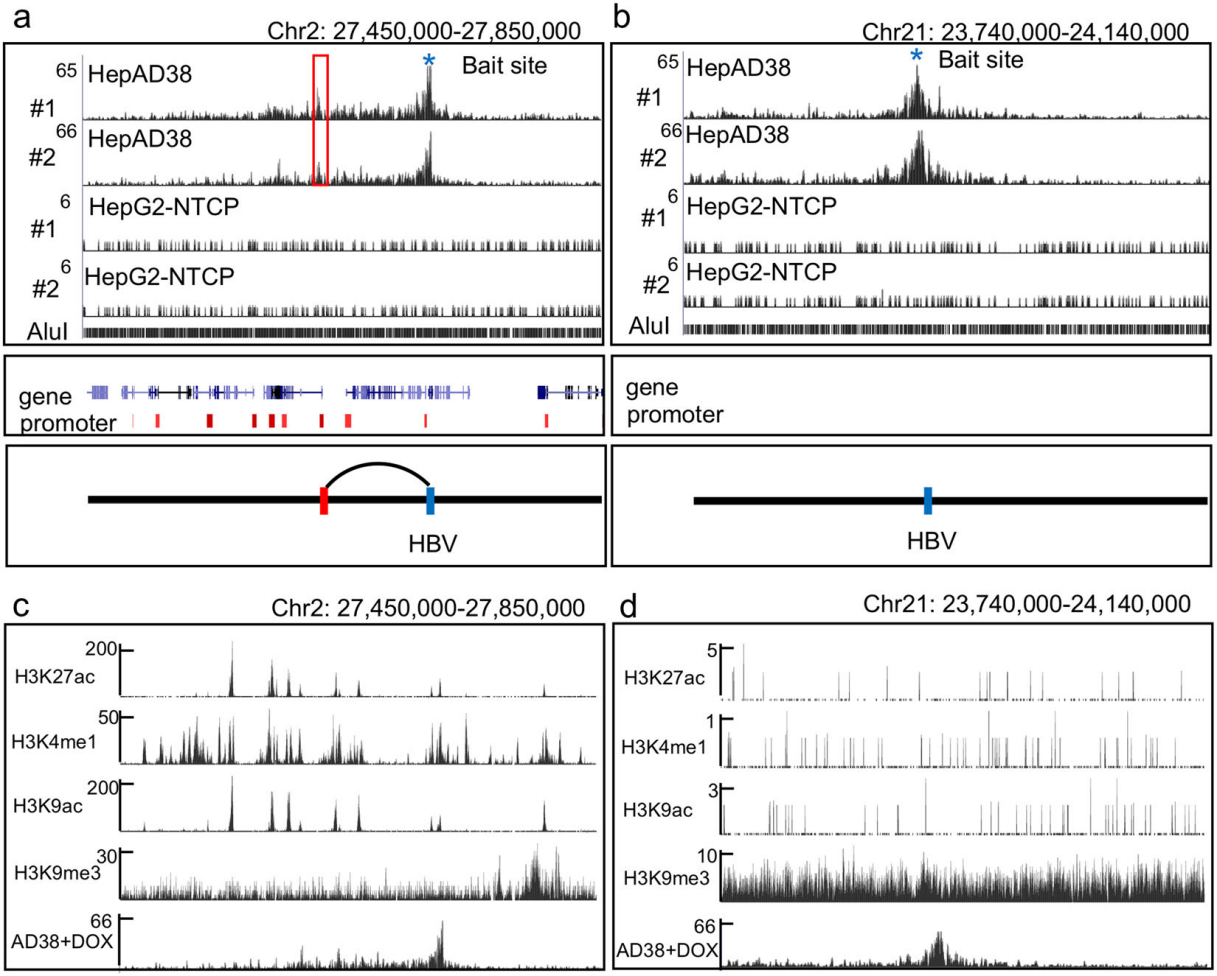
The genome organizations of integrated HBV DNA with human chromosomes. **a**, **b** Top: 3C-HTGTS interaction profiles of the integrated HBV DNA in HepAD38 cells with DOX and HepG2-NTCP cells infected by HBV. In chromosome 2 (**a**) or chromosome 21 (**b**). #1 using primer 1 datasets, #2 using primer 2 datasets visualization with UCSC Genome Browser (we used vertical viewing range setting for sequencing data display). Middle: the genes and promoters in that locale regions (promoter regions from GeneHancer Track of UCSC Genome Browser). Bottom: schematic representation of chromosome interactions of the integrated HBV DNAs with Human DNAs. **c**, **d** UCSC Genome Browser track showing integrated HBV DNA–host DNA contacts (using HepAD38 #2 3C-HTGTS sample), H3K27ac, H3K4me1, H3K9ac, and H3K9me3. The ChIP-seq data were from the ENCODE, generated by Bradley Laboratory, which were used HepG2 cells (we used vertical viewing range setting for sequencing data display).

Recent studies have focused on the HBV integration and its potential to drive carcinogenesis. Thus, we next investigated the contacts of integrated HBV DNA across the host genome in HepAD38 cell lines that have the known integrated HBV DNA segment. In particular, our data manifested a number of HBV DNA interactions with human chromosome 2 and chromosome 21 (Fig. 1d and Supplementary Fig. S2f right panel), all near the bait sites of HBV DNA integration^15^ (Supplementary Fig. S6c, d). Interestingly, and importantly, the integrated HBV DNA on chromosome 2 particularly forms a chromosome loop with host DNA, but not that on chromosome 21 (Fig. 6a, b and Supplementary Fig. S6a). To explore the potential mechanism of the distinct genome organization, we download previously published epigenetic datasets of HepG2 cells from ENCODE. In the region of HBV DNA-integrated sites on chromosome 2, H3K27ac, H3K9ac, and H3K4me1 modifications are enriched but H3K9me3 are not (Fig. 6c, d), whereas in integration site on chromosome 21, histone modifications H3K9me3 are enriched, but not H3K27ac, H3K9ac, H3K4me1. These results suggest integrated HBV DNAs can form chromatin loop structure with the cellular transcription active DNA regions.

### Transcriptional impact of HBV DNA–host DNA contacts

**Fig. 7:**
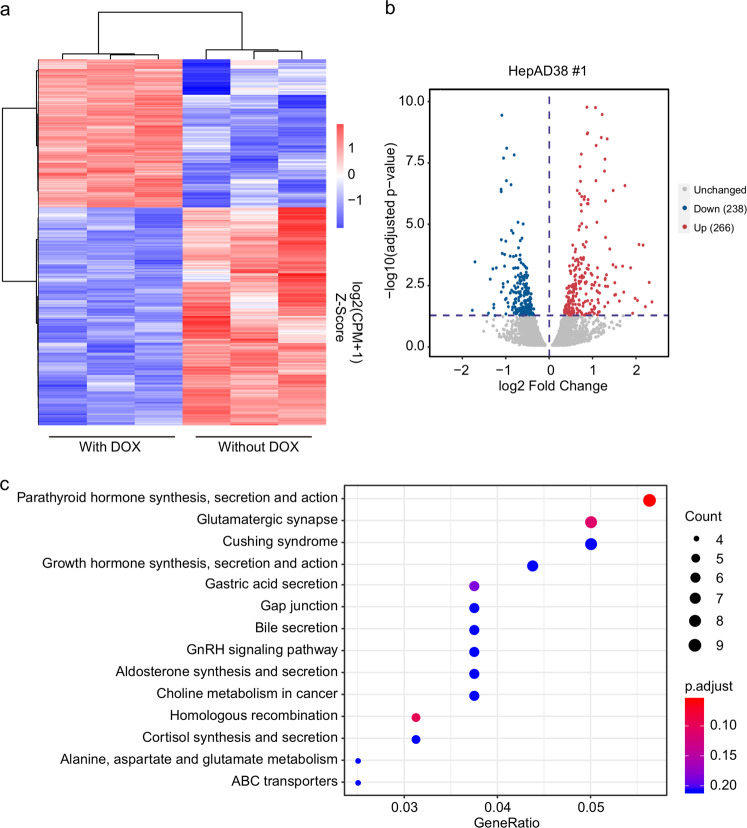
Impact of HBV DNA–host DNA contacts on cellular gene expression. **a** Heatmap of altered host gene expression upon HBV infection in DOX-withdrawed HepAD38 cells compared with HepAD38 cells with DOX. **b** Volcano plot showing the differential genes influenced by HBV infection that are overlapped with the HBV DNA contact regions (using HepAD38 #1 3C-HTGTS samples). **c** Gene enrichment analysis for differential genes simultaneously contacting with HBV DNA in HepAD38 #1 and HepAD38 #2 3C-HTGTS samples after HBV infection.

To investigate the potential role of HBV DNA–host DNA contacts on host gene expression, we performed RNA sequencing (RNA-Seq) analysis with HepAD38 cells 6 days after DOX withdrawal compared with HepAD38 cells with DOX. In total, 364 genes were significantly downregulated and 535 genes were upregulated in HBV-expressing HepAD38 cells (Fig. 7a). The pathway-enrichment analysis revealed that the genes affected by HBV expression are enriched in HIF-1 signaling pathway. To find the genes modulated by the HBV DNA–host DNA contacts, the comparative analysis of 3C-HTGTS and RNA-Seq suggested that HBV DNA–host DNA contacts are associated with the genes affected by HBV expression (Fig. 7b and Supplementary Fig. S7a-c). Then we performed the pathway-enrichment analysis using the genes associated with HBV contact and we found that those genes are also enriched in parathyroid hormone synthesis, secretion, and action pathway (Fig. 7c). In addition, we analyzed the impact of integrated HBV DNA–host DNA contacts on host gene expression and we found that most of the genes, those nearby the integrated HBV DNA on chromosome 2, were upregulated (Supplementary Fig. S7d). Taken together, our results indicate that most gene expression alterations, which are affected by HBV infection, are accompanied with HBV DNA–host DNA contacts.

## Discussion

Here we employed the 3C-HTGTS to investigate two types of HBV DNA interactions with host genome in different kinds of HBV infection models. The 3C-HTGTS technology coupling the 3C and HTGTS provides a high sensitivity with AluI digestion to have small chromatin fragments and linear amplification-mediated PCR to enlarge the weak signals, which would detect both the strong and weak regions for HBV DNA contacts with human genome. Based on this high-resolution chromatin structure mapping, our data demonstrated HBV DNA does not randomly position in host nucleus but instead preferentially contacts at active chromatin regions, which is consistent with the previous finding in Moreau et al.^8^. The HBV DNA–host DNA contacts specifically enriched with active H3K4me1 modification by KMT2C/D, which shares with HBV cccDNA mini-chromosome and strongly influences HBV transcription. In contrast, integrated HBV DNA forms chromatin loop with host DNA when the region of HBV DNA integrated is full of transcription active epigenetic marks.

Recently, overwhelming data suggest that genome is not randomly folded in interphase nucleus^16,17^. Each chromosome occupies a specific region of nucleus, which is known as chromosome territories^18^. At the sub-chromosome scale, the Hi-C data display a plaid pattern, suggesting the existence of two compartments (Compartment A/B). Compartment A regions contain high density of genes, H3K36me3 modifications, and DNase hypersensitive sites, exhibiting strong transcription activities, whereas compartment B regions overlap strongly with lamina-associated domain. Consistent with previous studies, our results demonstrated HBV DNA contacts preferentially at active chromatin regions of human genome with high resolution. We identified H3K4me1 modification by KMT2C/D is enriched both in host genome regions of HBV DNA contacts and in HBV cccDNA, which indicated HBV mini-chromosome shares the histone modifications with contacting human genomic regions. This shared modification is also required for HBV transcriptions. Our findings demonstrated both KMT2C and KMT2D knockdown markedly decreased HBV RNA expressions, which suggested a new epigenetic modification of HBV cccDNA for its transcription. It will be interesting to further investigate whether and how KMT2C/D are recruited to cccDNA mini-chromosome.

Although several studies have investigated HBV DNA can integrate into the human genome, contributing to genomic instability and hepatocarcinogenesis, the genome organization of this integrated HBV DNA remains unknown. Our study provided the primary evidence to investigate the spatial patterns of integrated HBV DNA within human genome by taking advantages of high sensitivity of 3C-HTGTS. Interestingly, the integrated HBV DNA particularly forms a chromatin loop with the host genome where active histone modifications are enriched. The integrated HBV DNA forms loop structure through contacting with host DNA with our high resolution of 3C-HTGTS data, it is indicating that there are some protein machinery maintaining the loop structure through binding with HBV DNA and cellular DNA (Supplementary Fig. S6B). Therefore, based on our 3C-HTGTS results, we hypothesize the spatial organization of HBV DNA with host chromatins in two different stages. First, when rcDNA is released into nucleoplasm and then transformed into cccDNA, the HBV minichromosomal cccDNA is distributed in human nucleus with contacts throughout host genome. In this stage, the HBV mini-chromosome (cccDNA) utilizes the cellular transcriptional and chromatin machinery for viral protein production and replication and starts to preferentially interact with host genome at active transcriptional regions. Second, when HBV DNA integrates into the host genome and forms the integrated HBV DNA, the integrated HBV DNA could further interact with host genome and form the chromatin loop structures. (Supplementary Fig. S8)

In conclusion, taking advantage of 3C-HTGTS in cell models of HBV infection, we obtained genome-wide organizations of HBV DNA and integrated HBV DNA within human genome. HBV DNA contacts host genome preferentially at active chromatin regions where the H3K4me1 is particularly enriched and required for HBV transcription. However, integrated HBV DNA can particularly organize chromatin loop structures that is sharply dependent on the local genome epigenetic environment.

## Methods

### Cell culture, HBV production, and infection

The HepAD38 cell line derives from HepG2 cells and contains the HBV genome (sub-type ayw) under tetracycline control. HepAD38 cells were maintained in Dulbecco’s modified Eagle medium (DMEM)/F-12 with 10 % fetal bovine serum (FBS). HepG2-NTCP cells (AC12 clone) derive from HepG2 cells overexpressing human sodium taurocholate co-transporting polypeptide (NTCP). HepG2-NTCP cells are grown in DMEM with 10% FBS.

For virus production, HepAD38 cells were grown in Williams E medium supplemented with 10% FBS. HBV particles were concentrated from the clarified supernatant through overnight precipitation in 5% PEG 8000, followed by centrifugation at 4 °C (60 min at 5292 × *g*). Titers of enveloped DNA-containing viral particles were determined by qPCR quantification of viral RC-DNA using RC primers. For infection, only enveloped DNA-containing viral particles (vp) were taken into account to determine the multiplicity of infection (MOI). AC12s were infected for 6 days with normalized amounts of virus at an MOI of 500 vp/cell.

### Quantitative reverse-transcriptase PCR

Total RNA was prepared using TRIzol reagent (Invitrogen). RNA (1000 ng) was retro-transcribed using random primers and reverse transcriptase. Reverse-transcriptase qPCR experiments were carried out as described. For relative quantifications, actin was used as a reference gene. Values were calculated according to the ΔCt quantification method with ΔCt = Ct HBV − Ct actin. Results demonstrated the mean values of at least three independent experiments. SEM are indicated. *P*-values were determined by Mann–Whitney test. The primers of HBV total RNA-F and HBV total RNA-R were used to amplify all HBV transcripts (pgRNA as well as the 2.4 and 2.1 kb mRNA). pgRNA F and pgRNA R primers were specific for pgRNA (Supplementary Table S1).

### Quantification of cccDNA

For DNA isolation, cells were lysed using lysis buffer containing proteinase K (200 μg/mL) and then incubated for 4 h at 55 °C. Total DNA were isolated according to standard genomic DNA isolation procedure. Then digested 500 ng DNA with plasmid-safe ATP-dependent DNase for 8 h at 37 °C to allow removal of linear genomic DNA and HBV replication intermediates (rcDNAs, single-strand DNAs, linear double-strand DNAs), and cccDNA was amplified using cccDNA primers (Supplementary Table S1).

### 3C-HTGTS library preparation

The 3C libraries were generated as previously described. Briefly, ten million cells were cross-linked with 1% (v/v) formaldehyde for 10 min at room temperature, followed by quenching with glycine at a final concentration of 125 mM. Cells were lysed in 50 mM Tris-HCl pH 7.5, containing 150 mM NaCl, 5 mM EDTA, 0.5% NP-40, 1% Triton X-100, and protease inhibitors. Nuclei were digested with 500 units of *Alu*I restriction enzyme at 37 °C overnight, followed by ligation under dilute conditions overnight. Crosslinks were reversed and samples were treated with Proteinase K and RNase A prior to DNA precipitation. The 3C libraries were sonicated for 30 s ON and 60 s OFF for two cycles on a Bioruptor Sonicator. Sonicated DNA was linearly amplified with a biotinylated primer. The biotin-labeled single-stranded DNA products were enriched with streptavidin C1 beads, and 3′-ends were ligated with the bridge adaptor containing a six-nucleotide overhang. The adaptor-ligated products were amplified by a nested primer and an adaptor-complementary primer. The primers used for making 3C-HTGTS libraries are listed in Supplementary Table S2. Data were plotted for comparison after normalizing junction from each experimental 3C-HTGTS library by random selection to the total number of genome-wide junctions recovered from the smallest library in the set of libraries being compared.

### Chromatin immunoprecipitation

ChIP analysis was performed as previously described with the antibodies listed in Supplementary Table S3. Briefly, 5 μg of specific antibodies was bound to Dynal magnetic secondary beads for 6 h. After washing, sonicated chromatin from WT or KMT2C/D KD cell was incubated with the bead-bound antibody overnight.

### RNA sequencing

RNA purity was checked using the kaiaoK5500®Spectrophotometer (Kaiao, Beijing, China). RNA integrity and concentration was assessed using the RNA Nano 6000 Assay Kit of the Bioanalyzer 2100 system (Agilent Technologies, CA, USA). A total amount of 2 μg RNA per sample was used as input material for the RNA sample preparations. Sequencing libraries were generated using NEBNext® UltraTM RNA Library Prep Kit for Illumina® (#E7530L, NEB, USA) following the manufacturer’s recommendations and index codes were added to attribute sequences to each sample. Briefly, mRNA was purified from total RNA using poly-T oligo-attached magnetic beads. Fragmentation was carried out using divalent cations under elevated temperature in NEBNext First Strand Synthesis Reaction Buffer. First-strand cDNA was synthesized using random hexamer primer and RNase H. Second-strand cDNA synthesis was subsequently performed using buffer, dNTPs, DNA polymerase I, and RNase H. The library fragments were purified with QiaQuick PCR kits and elution with EB buffer, then terminal repair, A-tailing, and adapter added were implemented. The aimed products were retrieved and PCR was performed, then the library was completed.

### Data analysis

#### 3C-HTGTS analysis

Pair-end illumina sequencing fastq data were remove adaptor and low-quality reads with cutadapt^19^ and then demultiplexing with fastq-multx^20^ (-m 0), and pair-end reads without enzyme site or bait site were filtered (enzyme site were filtered with trimLinker, one tools form ChIA-PET2^21^, with parameters -t 12 -m 2 -k 1 -l 16). The R2 end of the remaining reads were mapping to human genome hg19 with bowtie2^22^ (“--very-sensitive-local -L 30 --score-min G,20,8); The mapping reads were filtered the duplicated reads, self-ligation reads, relegation reads and dumped reads. For visualization, we convert the final bam files into bigwig file with bamCoverage^23^ (--binSize 10 --normalizeUsing RPKM), and then upload the bigwig file to UCSC genome browser^24^. For the visualization in Fig. 6, supplementary Fig S6, to get a more accurate view of the contacts, we also normalized the bias caused by restriction sites with 4C-ker^25^ and the significant interaction was called like Kim et al.^26^; briefly, we called the significant interaction with bdgpeakcall subcommand form MACS2^27^ (-c 20 -g 3000) and the consistent peak in #1 and #2 were used. The coloration analysis of histone markers and 3C-HTGTS data was performed by multiBigwigSummary^23^ (BED-file) for each chromosome separately.

#### Regulatory elements enrichment analysis

The coordinates of LaminB1 (track: laminB1Lads), CpG island (track: cpgIslandExt), and TSS (track: hg19_refGene, ±3.5 kb around the TSS) were download form UCSC^24^. The enhancer file was download from the Encode^28^ (file: hepatocytes-DS32057A.peaks.fdr0.01.hg19.bed). To detect the significance, we used a bootstrap method like Moreau et al.^29^. For a sample with N HBV contact reads, first, we extend each read to 7 kb centered on the start site of each read, then calculating the proportion of the extend reads overlapping with functional elements (sign as *P*_HBV_). Second, to get a random contact dataset considering the bias of enzyme sites, we random N *Alu*I sites, extend them to 7 kb centered on these enzyme sites and calculate the proportion of extend random reads overlapping with functional elements. Further, this procedure was reiterated 1000 times. Finally, the *P*-value was computed by calculating the probability by which *P*_HBV_ is from the 1000 random contact proportion.

#### RNA-seq analysis

RNA-seq data were sequenced on illumina with three replicates in pair-ended 150 bp mode. Fastq files removed adaptor and low-quality reads with cutadapt^19^, then the remaining reads were mapped to human genome GRCh37 with hisat2^30^, htseq-count^31^ was used to quantify gene counts with annotation file from GENCODE, and DESeq2^32^ were used to perform gene differential analysis. For heatmap, we select the false discovery rate < 0.05 as the cut-off. Enrichment pathway were performed with clusterProfiler^33^. To find the genes effected by the HBV cccDNA, we selected the genes overlapped with the 3C-HTGTS reads, then visualized with the volcano plot.

#### Software

The following softwares were used: cutadapt v1.15 (https://cutadapt.readthedocs.io/en/stable/), FastQC v0.11.6 (https://www.bioinformatics.babraham.ac.uk/projects/fastqc/), ChIA-PET2 v0.9.3 (trimLinker program: https://github.com/GuipengLi/ChIAPET2), Bowtie2 v2.2.8 (http://bowtie-bio.sourceforge.net/bowtie2/index.shtml), samtools v1.6 (https://github.com/samtools/samtools)^34^, BEDTools v2.26.0 (https://bedtools.readthedocs.io/)^35^, UCSC Genome Browser (https://genome.ucsc.edu/index.html), deeptools v3.13 (https://deeptools.readthedocs.io/en/develop/), Bioconductor v3.6 (DESeq2, clusterProfiler packages:https://www.bioconductor.org/), R v3.5.0 (RColorBrewer, ggplot2 packages: https://cran.r-project.org/), UCSC utilities (http://hgdownload.soe.ucsc.edu/admin/exe/), fastq-multx V1.3.1 (https://github.com/brwnj/fastq-multx), hisat2 V2.1.0 (http://ccb.jhu.edu/software/hisat2/)4C-ker (https://github.com/rr1859/R.4Cker), and htseq-count (https://htseq.readthedocs.io/en/master/count.html).

## Supplementary information

Supplementary Information

## Supplementary information

**Supplementary Information** accompanies the paper at (10.1038/s41421-020-00218-1).

## References

[1] Trepo, C., Chan, H, L. Y. & Lok, A. Hepatitis B virus infection. *Lancet* **384**, 2053–2063 (2014). 10.1016/S0140-6736(14)60220-8 24954675

[2] Dienstag, J. L. Hepatitis B virus infection. *N. Eng. J. Med*. **359**, 1486–1500 (2008). 10.1056/NEJMra0801644 18832247

[3] Tong S, Revill P (2016). Overview of hepatitis B viral replication and genetic variability. J. Hepatol..

[4] Bock CT (2001). Structural organization of the hepatitis B virus minichromosome. J. Mol. Biol..

[5] Tropberger P (2015). Mapping of histone modifications in episomal HBV cccDNA uncovers an unusual chromatin organization amenable to epigenetic manipulation. Proc. Natl Acad. Sci. USA.

[6] Pollicino T (2006). Hepatitis B virus replication is regulated by the acetylation status of hepatitis B virus cccDNA-bound H3 and H4 histones. Gastroenterology.

[7] Ren JH (2018). SIRT3 restricts hepatitis B virus transcription and replication through epigenetic regulation of covalently closed circular DNA involving suppressor of variegation 3-9 homolog 1 and SET domain containing 1A histone methyltransferases. Hepatology.

[8] Moreau P (2018). Tridimensional infiltration of DNA viruses into the host genome shows preferential contact with active chromatin. Nat. Commun..

[9] Jain S, Ba Z, Zhang Y, Dai HQ, Alt FW (2018). CTCF-binding elements mediate accessibility of RAG substrates during chromatin scanning. Cell.

[10] Sun Y, Qi Y, Peng B, Li W (2017). NTCP-reconstituted in vitro HBV infection system. Methods Mol. Biol..

[11] Ladner, S. K. et al. Inducible expression of human hepatitis B virus (HBV) in stably transfected hepatoblastoma cells: a novel system for screening potential inhibitors of HBV replication. *Antimicrob. Agents Chemother*. **41**, 1715–1720 (1997). 10.1128/aac.41.8.1715 PMC163991 9257747

[12] Zhang W (2017). PRMT5 restricts hepatitis B virus replication through epigenetic repression of covalently closed circular DNA transcription and interference with pregenomic RNA encapsidation. Hepatology.

[13] Dawkins JB (2016). Reduced expression of histone methyltransferases KMT2C and KMT2D correlates with improved outcome in pancreatic ductal adenocarcinoma. Cancer Res..

[14] Local A (2018). Identification of H3K4me1-associated proteins at mammalian enhancers. Nat. Genet..

[15] Li, M., Sohn, J. I. & Seeger, C. Distribution of hepatitis B virus nuclear DNA. *J. Virol*. **92**, JVI.01391-17 (2018). 10.1128/JVI.01391-17 PMC5730781 29046450

[16] Bickmore WA (2013). The spatial organization of the human genome. Annu. Rev. Genomics Hum. Genet..

[17] Sexton T (2012). Three-dimensional folding and functional organization principles of the *Drosophila* genome. Cell.

[18] Bolzer A (2005). Three-dimensional maps of all chromosomes in human male fibroblast nuclei and prometaphase rosettes. PLoS Biol..

[19] Martin M (2011). Cutadapt removes adaptor sequence form high-throughput sequencing reads. EMBnet. J..

[20] Aronesty E (2013). Comparison of sequencing utility programs. Open Bioinformatics J..

[21] Li G, Chen Y, Snyder MP, Zhang MQ (2017). ChIA-PET2: a versatile and flexible pipeline for ChIA-PET data analysis. Nucleic Acids Res..

[22] Langmead B, Salzberg SL (2012). Fast gapped-read alignment with Bowtie 2. Nat. Methods.

[23] Ramirez F, Dundar F, Diehl S, Gruning BA, Manke T (2014). deepTools: a flexible platform for exploring deep-sequencing data. Nucleic Acids Res..

[24] Karolchik D (2014). The UCSC Genome Browser database: 2014 update. Nucleic Acids Res..

[25] Raviram R (2016). 4C-ker: a method to reproducibly identify genome-wide interactions captured by 4C-Seq experiments. PLoS Comput. Biol..

[26] Kim KD (2020). Epigenetic specifications of host chromosome docking sites for latent Epstein-Barr virus. Nat. Commun..

[27] Zhang Y (2008). Model-based analysis of ChIP-Seq (MACS). Genome Biol..

[28] Consortium EP (2012). An integrated encyclopedia of DNA elements in the human genome. Nature.

[29] Moreau P (2018). Tridimensional infiltration of DNA viruses into the host genome shows preferential contact with active chromatin. Nat. Commun..

[30] Kim D, Langmead B, Salzberg SL (2015). HISAT: a fast spliced aligner with low memory requirements. Nat. Methods.

[31] Anders S, Pyl PT, Huber W (2015). HTSeq-a Python framework to work with high-throughput sequencing data. Bioinformatics.

[32] Love MI, Huber W, Anders S (2014). Moderated estimation of fold change and dispersion for RNA-seq data with DESeq2. Genome Biol..

[33] Yu G, Wang LG, Han Y, He QY (2012). clusterProfiler: an R package for comparing biological themes among gene clusters. OMICS.

[34] Li H (2009). The Sequence Alignment/Map format and SAMtools. Bioinformatics.

[35] Quinlan AR (2014). BEDTools: the Swiss-army tool for genome feature analysis. Curr. Protoc. Bioinformatics.

