# Supplementary material for: 3D landscape of Hepatitis B virus interactions with human chromatins
Source: Cell Discov. 2020 Dec 29;6:95. doi: 10.1038/s41421-020-00218-1 (PMC7769987; doi:10.1038/s41421-020-00218-1)
Supplement: Supplementary file 1 — Supplementary Information [file 41421_2020_218_MOESM1_ESM.doc]

**Supplementary** **Information**

**3D landscape of Hepatitis B virus interactions with the chromatin of human cells**

Yang B et al.

Supplementary Figs.1-8

Supplementary Fig. S1. Schematic representation of the 3C-HTGTS methods for studying chromosomal interactions of HBV DNA contacts with human genome.

a. The figure shows the outline of the 3C-HTGTS. 1. Cross-linking by formaldehyde and digestion with the restriction enzyme (AluI), then the “hairballs” of cross-linked DNA are created. 2. Chromatin is diluted and re-ligated to fuse the ends of DNA fragments present in the same “hairballs”. The ultimate outcome of this ligation event is large DNA circles. 3. The smaller DNA fragments are generated by sonication, and the cross-links are removed by salt and heating. 4. Using a biotin labeled primer to amplify the interested DNA fragments by linear amplification-mediated (LAM) PCR, then purify the PCR products through the streptavidin beads. 5. The purified PCR products are ligated to bridge adapters using DNA ligase. 6. Two rounds of PCR amplification to make it useable for high-throughput sequencing. b. The figure shows that the principle of primer design. The nested primer is designed with 50-120bp base pairs of neighboring the restriction sire; the biotin primer is designed with 10-50bp base pairs of neighboring the nested primer. c. The restriction enzyme test. The enzymes are marked by numbers. The DNA ladder is DL2000, the digestion time are different from 30min to overnight. d. The primer designed for HBV 3C-HTGTS.

Supplementary Fig. S2. Distribution of HBV DNA-host DNA contacts throughout the Human genome.

a-b. Model of HBV infection cell system(a) and inducible expression of HBV cell system(b). The level of HBV RNAs and cccDNA were detected at the 0 day, 1 day and 6 days when HBV infected HepG2-NTCP or HepAD38 cell withdraws DOX. c-d. HBV transcription level and cccDNA level were analyzed by RT-PCR in HBV infected HepG2-NTCP cells (c) and in HepAD38 cells (d). e. The reads we got from different HBV cell model. f. The HBV DNA-host DNA contacts distribution density in different chromosome. HBV infected HepG2-NTCP cells using primer 1 and HepAD38 with DOX cells using primer 1.

Supplementary Fig. S3. HBV DNA-host DNA contacts are preferential at active chromatin regions.

a-b. Heatmap of HBV DNA-host DNA contacts enrichment for different histone marks in HepAD38 with DOX sample using primer #1(a) and primer #2(b). c-d. Heatmap of HBV DNA-host DNA contacts enrichment for different histone marks in different samples, c figure using 250kb bin, d figure using 50 kb bin. Signals for the heatmap are represented by spearman correlation. Histone marks dataset were from HepG2 cells.

Supplementary Fig. S4. Histone posttranslational modifications H3K4me1 in HBV cccDNA.

a. The H3K4me1 modification in cccDNA mapped by ChIP-qPCR in HepAD38 cells. b. The H3K4me1 recruitment was analyzed by ChIP-qPCR in HepG2-NTCP cells by HBV infected. c. The ChIP assay performed in human liver samples. Error bars indicate SEM and an asterisk a significant statistical difference (** p<0.01, *** p<0.001).

Supplementary Fig. S5. KMT2C/D are required for HBV transcription.

a-b. The effect of KMT2C/D knockdown to KMT2C/D RNA level by siRNA transfection. c. The effect of KMT2C knockdown to KMT2C protein level. d. The quantitative statistics for the western blot result by Image J. e. Model of siRNA transfection experiments in HepG2-NTCP cells by HBV infection. The HepG2-NTCP cells were treated by siRNA before HBV infection 2.5 days, and the level of HBV RNAs and HBeAg were detected after HBV infection 3.5 days. f-g. HBV transcription was analyzed by RT-qPCR in HepG2-NTCP cells which are treated by siRNA before HBV infection 2.5 days. h. HBeAg was analyzed by ELISA in HepG2-NTCP cells which are treated by siRNA before HBV infection 2.5 days. Error bars indicate SEM and an asterisk a significant statistical difference (* p<0.05).

Supplementary Fig. S6. The interactions of integrated HBV DNA with other Human chromosome regions.

a. The peak, genes, promoter and Hi-C result in the HBV DNA integration region on chromosome 2 (27,450,000-27,850,000). The Hi-C (generated by Dekker Laboratory) heatmap of HepG2 cells was generated from the public available website (<http://promoter.bx.psu.edu/hi-c/view.php>) (Wang et al., 2018), which we used hg38 and CAD and CCDC121 genes to locate the corresponding chromosome location of the 3C-HTGTS track (We used vertical viewing range setting for sequencing data display). b. the top panel shows the primers sites, the AluI restriction enzyme sites, and the CMV promoter site in the HepAD38 cells. The rest panel shows the different scenarios. 1. The CMV promoter and HBV DNA both contact with cellular DNA, in this scenario we can get contact from the results of primer 1/2. 2. Only the HBV DNA contacts with cellular DNA, in this scenario, we also can get the contact from the results of primer1/2. 3. Only the CMV promoter contacts with cellular DNA, in this scenario, we cannot get contact from the result of primer2, because if there is no contact between HBV DNA and cellular DNA, the HBV DNA will be far away from the cellular DNA after the treatment of digestion and dilution. c. The top panel was the HBV DNA-host DNA contacts distribution (normalized with 4C-ker) in this region. The middle panel was the PCR result; the red arrows indicated the PCR products that had the integrated HBV DNA, and the magnification track above the PCR products was normalized by RPKM with bamCoverage. The bottom panel was the PCR sequencing result that blast with the human genome (chromosome21). We used a vertical viewing range setting for sequencing data display. d. The top panel was the HBV DNA-host DNA contacts distribution (normalized with 4C-ker) in this region (We used vertical viewing range setting for sequencing data display). The bottom panel was the PCR results; the red arrows indicated the PCR products that had the integrated HBV DNA (chromosome2).


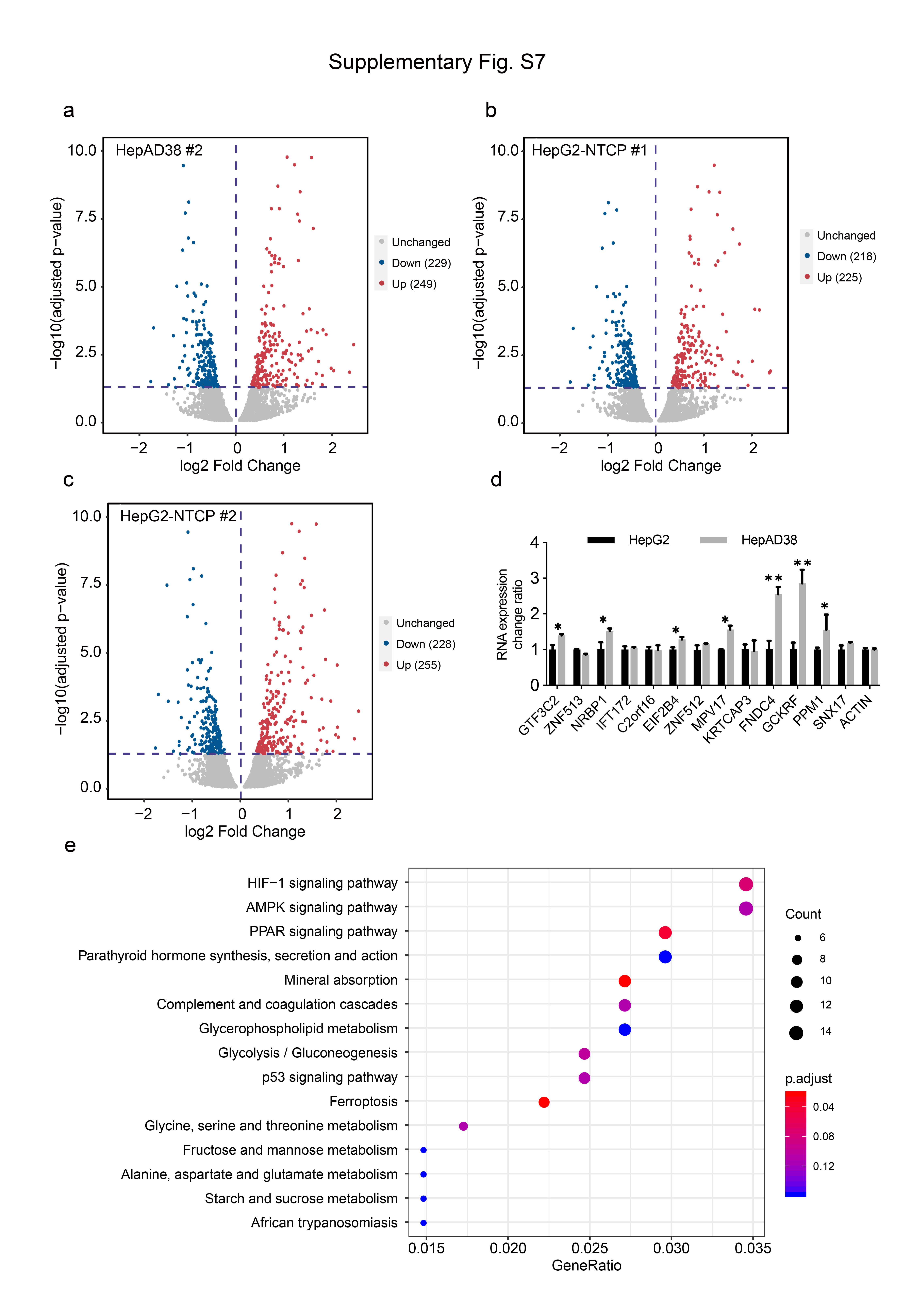


Supplementary Fig. S7. Impact of HBV DNA-host DNA contacts on cellular gene expression.

a-c. Volcano plot to show the differential genes effected by HBV infection (HepAD38 without DOX compare with HepAD38 with DOX) that are overlapped with the HBV DNA contact regions. d. The gene expression level affected by the HBV DNA integration on chromosome 2. e. Gene enrichment analysis for differential genes after HBV infection.

Supplementary Fig. S8. The model of the spatial organization of HBV DNAs in nuclear.

Left panel show the HBV life cycle and four kind of HBV DNA in nuclear. Right panel show in different level, how HBV DNA are organized. In chromosome territory level, the HBV DNA contact with entire genome. In compartment level, the HBV DNA-host DNA contacts are enriched in the transcription active regions. In the chromatin loops level, the integrated HBV DNA form chromosome loop with host DNA. Based on this results, we hypothesize the spatial organization of HBV DNA with host chromatins in two different stage. First, when rcDNA is released into nucleoplasm and then transformed into cccDNA, the HBV minichromosomal cccDNA is distributed in human nucleus with contacts throughout host genome. In this stage, the HBV minichromosome (cccDNA) utilizes the cellular transcriptional and chromatin machinery for viral protein production and replication and starts to preferentially interact with host genome at active transcriptional regions. Second, when HBV DNA integrates into the host genome and forms the integrated HBV DNA, the integrated HBV DNA could further interact with host genome and form the chromatin loop structures.

Supplementary Table S1

The list of primers used in PCR

| Primer name | Sequence |
| --- | --- |
| DNAJC5Gf | CGGTATCATCCCGACAAGAATC |
| DNAJC5Gr | TCTTAGAGTCGCTCAGTATGGC |
| UCNf | CAACCCTTCTCTGTCCATTGAC |
| UCNr | CGAGTCGAATATGATGCGGTTC |
| GTF3C2f | GGCTTCCGTAGAGATGTCATTAC |
| GTF3C2r | GCCTTTTCGTGTCCTACCACC |
| ZNF513f | GTGACAAACCTTTTCGGTGTAGC |
| ZNF513r | CAGTGGCCCGTGGTATAGG |
| NRBP1f | ACGATGAATGAAAAGGCATGGA |
| NRBP1r | GCCAATCTTGATGAGTCCGTT |
| IFT172f | CTGTGATCGGAAAATTGTAGCCT |
| IFT172r | GCCTGTCATAACTTCCTAGCAC |
| C2orf16f | CAGAGCCAACTAGGAAGTTCCC |
| C2orf16r | ACCCTGGTGCTAATTCCACAG |
| EIF2B4f | CTCTCACCTACCCCAGTACAG |
| EIF2B4r | ACGAAGCAGGGCAATACACC |
| ZNF512f | CCACTTCGGGACCCACAAC |
| ZNF512r | GATGCAGACGATGAACCACTT |
| SLC30A3f | CACCCGCACCATGACCTTT |
| SLC30A3r | AAGGCCATTAACAGGTTGGCA |
| TRIM54f | ATCGTGCAGGCATGAGGTTG |
| TRIM54r | CCTCGCACATGAGGTGCTG |
| MPV17f | CCCGTGGAAAGTACAGGTCC |
| MPV17r | ATGAACCGATCCAAAACCTTGT |
| KRTCAP3f | TACACCGTAGCCAATGTCATCT |
| KRTCAP3r | CAACGGACAAGAGCAGGTTC |
| FNDC4f | CACTTCCGAACTCTCAAGGGT |
| FNDC4r | GCAGAACAGCCCAATTACAGC |
| GCKRf | AGAGACTCTACAGCGAATCCAT |
| GCKRr | ACACCGACATGAGGAATGCC |
| PPM1Gf | GAGCTGCTGACACGCTACG |
| PPM1Gr | TTCCCTAGTTGAGTCCTCAGG |
| SNX17f | GGTCAACGTGCTAACTTCAGA |
| SNX17r | GCTCCATCCTCTTTTTCTCGAAC |
| HBV 3.5 kb transcripts | GAGTGTGGATTCGCACTCC |
| HBV 3.5 kb transcripts | GAGGCGAGGGAGTTCTTCT |
| total HBV transcripts | TCACCAGCACCATGCAAC |
| total HBV transcripts | AAGCCACCCAAGGCACAG |
| HBV cccDNA F | CCTACTGTTCAAGCCTCCAAGC |
| HBV cccDNA R | CCTGAGTGCAGTATGGTGAGG |
| enhancer 1-core f | GCGTTGATGCCTTTGTATGC |
| enhancer 1-core r | GGGTTGCGTCAGCAAACAC |
| enhancer 2 f | TCTTACATAAGAGGACTCTTGGAC |
| enhancer 2 r | CCTAATCTCCTCCCCCAACTC |
| S1 Promoter f | CCTTATTATCCAGAACATCTAGTTAATC |
| S1 Promoter r | ACCCACAAAATGAGGCGCTATG |
| S2 Promoter f | CCAACAAGGACACCTGGCC |
| S2 Promoter r | GGATGAGTGTTTCTCAAAGGTGGAG |
| HBV DNA1 F | CTGGACTATCAAGGTATGTTGCCC |
| HBV DNA1 R | CAATTTCCGTCCGAAGGTTTGGTAC |
| HBV DNA2 F | GCCAAGTCTGTACAGCATCTTGAG |
| HBV DNA2 R | GTGGCAAGGACCCATAACATCC |
| HBV DNA4 F | CCATGGCTGCTAGGCTGTG |
| HBV DNA4 R | AACGGCAGACGGAGAAGGG |
| HBV DNA6 F | GGAGCTACTGTGGAGTTACTCTCG |
| HBV DNA6 R | GCTTGCCTGAGTGCAGTATGG |
| HBV DNA9 F | TCCACCAAACTCTGCAAGATCCC |
| HBV DNA9 R | TCTCCATGTTCAGCGCAGGG |
| HBV DNA10 F | CCTCACAATACCGCAGAGTCTAG |
| HBV DNA10 R | CCAGCGATAACCAGGACAAGTTG |
| Chr22-1 | GCATTAACTGAGGTGGCGGG |
| Chr22-2 | CAGGGAAATGGTGGCCACC |
| Chr22-3 | GGAAAGTCCAAGATCAAGGTGCC |
| Chr22-4 | CAGAGTCTGAGGTTAACACAGTGG |
| Chr22-5 | GAGAGGGAGACAGTGACTTGGG |
| Chr22-6 | CTCAGCCACAACATCCTCGAG |
| Chr22-7 | CGGGGTTTCACCATGTTGGC |
| Chr22-8 | CTGCATTCTGGGTGACTTCC |
| Chr22-9 | CACGGTGTCACTCTGTCACC |
| Chr22-10 | CGCAGTAACACACATGAGCTG |
| Chr22-11 | GCCAAAGGTGCCAGCTCCC |
| Chr22-12 | TGGCTGTCTCCAAGGAGCCC |
| chr2-1 | GATCACACAGCTGGACAAATTGGG |
| chr2-2 | GAAAGAATGAATGAGAAAGCGAACGG |
| chr2-3 | GCCTGACCAACCGCGAGG |
| chr2-4 | GGTGAACACCACCACCCGGG |
| chr2-5 | AGGAGTTTGAGACCAGCCTGG |
| chr2-6 | GAGAGATGCACTAACAACTGGGG |
| chr2-7 | GTGTTCCTGTGTCTGTTCTTCCCTC |
| chr2-8 | CCCAATTAGGAAGTAGGGTACC |
| chr2-9 | GTTGCGCTCCGGTCGCATAAG |
| chr2-10 | GCTGTATGATGAACATGGAGAACGG |
| chr2-11 | GATTACAGACGTGCGCTAGCAC |

Supplementary Table S2

The list of primers used for making 3C-HTGTS libraries

| Primer name | Sequence |
| --- | --- |
| Adapter-upper-6N | CTGGAGTTCAGACGTGTGCTCTTCCGATCTNNNNNN-NH2 |
| Adapter-lower-NH2 | /5Phos/AGATCGGAAGAGCACACGTCTGAACTCCAG-NH2 |
| P5-I5 | AATGATACGGCGACCACCGAGATCTACACACACTCTTTCCCTACACGACGC |
| P7-I7 | CAAGCAGAAGACGGCATACGAGAT |
| Biotin primer 2# | biotin- TTCCCGAGATTGAGATCTTCTGCGACGCGG |
| Biotin primer 1# | biotin- GGGGCAGAATCTTTCCACCAGCAATCCTC |
| Nested primer 2#-1 | ACACTCTTTCCCTACACGACGCTCTTCCGATCTGCCTAAGTTCTTCTTCTAGGGGACCTGCCTCGTCG |
| Nested primer 2#-2 | ACACTCTTTCCCTACACGACGCTCTTCCGATCTTGGTCAGTTCTTCTTCTAGGGGACCTGCCTCGTCG |
| Nested primer 1#-1 | ACACTCTTTCCCTACACGACGCTCTTCCGATCTCACTGTCCAGCCTTCAGAGCAAACACCGCAAATCC |
| Nested primer 1#-2 | ACACTCTTTCCCTACACGACGCTCTTCCGATCTATTGGCCCAGCCTTCAGAGCAAACACCGCAAATCC |
| Nested primer 2#-3 | ACACTCTTTCCCTACACGACGCTCTTCCGATCTttgactGTTCTTCTTCTAGGGGACCTGCCTCGTCG |
| Nested primer 2#-4 | ACACTCTTTCCCTACACGACGCTCTTCCGATCTggaactGTTCTTCTTCTAGGGGACCTGCCTCGTCG |
| Nested primer 1#-3 | ACACTCTTTCCCTACACGACGCTCTTCCGATCTtgacatCCAGCCTTCAGAGCAAACACCGCAAATCC |
| Nested primer 1#-4 | ACACTCTTTCCCTACACGACGCTCTTCCGATCTggacggCCAGCCTTCAGAGCAAACACCGCAAATCC |

Supplementary Table S3

| Name | Company | Catalog |
| --- | --- | --- |
| Mono-Methyl-Histone H3 (Lys4) (D1A9) | Cell signaling technology | 5326 |
| Rabbit (DA1E) mAb IgG | Cell signaling technology | 3900 |
| Histone H3 | Cell signaling technology | 4499 |
| Anti-GAPDH mAb-HRP-DirecT | Bei jing Xin Hua Lv Yuan science and Technology | M171-7 |
| MLL2(2E1) | Santa Cruz Biotechnology | sc-293217 |

The list of antibody
